# Total Synthesis and Antimicrobial Activity of a Natural Cycloheptapeptide of Marine Origin

**DOI:** 10.3390/md8082384

**Published:** 2010-08-19

**Authors:** Rajiv Dahiya, Hemendra Gautam

**Affiliations:** Department of Pharmaceutical Chemistry, NRI Institute of Pharmacy, Bhopal 462 021, Madhya Pradesh, India; E-Mail: rajivdahiya77@rediffmail.com

**Keywords:** marine natural product, stylisin 2, Stylissa caribica, peptide synthesis, antimicrobial activity

## Abstract

The present study deals with the first total synthesis of the proline-rich cyclopolypeptide stylisin 2 via a solution phase technique by coupling of the Boc-l-Pro-l-Ile-l-Pro-OH tripeptide unit with the l-Phe-l-Pro-l-Pro-l-Tyr-OMe tetrapeptide unit, followed by cyclization of the resulting linear heptapeptide fragment. The chemical structure of the finally synthesized peptide was elucidated by FTIR, ^1^H/^13^C-NMR and FAB MS spectral data, as well as elemental analyses. The newly synthesized peptide was subjected to antimicrobial screening against eight pathogenic microbes and found to exhibit potent antimicrobial activity against *Pseudomonas aeruginosa*, *Klebsiella pneumoniae* and *Candida albicans*, in addition to moderate antidermatophyte activity against pathogenic *Trichophyton mentagrophytes* and *Microsporum audouinii* when compared to standard drugs—gatifloxacin and griseofulvin.

## 1. Introduction

During past years, natural products derived from marine sponges have played a crucial role in the pharmaceutical research as biomedically useful agents or as lead compounds for drug development. Among them, bioactive cyclopeptides with unique structures have emerged as vital organic congeners which may prove better candidates to overcome the problem of widespread increase of microbial resistance towards conventional drugs [1]. Diverse biological activities possessed by marine sponge-derived natural cyclic peptides include antimicrobial, cytotoxic, anti-HIV, nematocidal, anti-inflammatory, serine protease and protein tyrosine phosphatase inhibitory activity [2–8]. Stylisin 2, a natural cyclic heptapeptide has been isolated from the Jamaican sponge *Stylissa caribica* and its structure was elucidated on the basis of 1D and 2D NMR data (^1^H, ^13^C, COSY, HMQC, HMBC, ROESY), followed by determination of absolute configuration of amino acids by Marfey’s analysis [9].

Prompted by the pharmacological properties of proline-rich cyclopeptides [10–13] as well as to obtain a natural bioactive peptide in good yield and in continuation of our efforts toward synthesizing natural cyclopeptides [14–27], the present study was aimed at the first total synthesis of stylisin 2 (**8**) using solution phase techniques. The synthesized cyclooligopeptide was also subjected to antibacterial and antifungal screening.

## 2. Results and Discussion

**Scheme 1:**
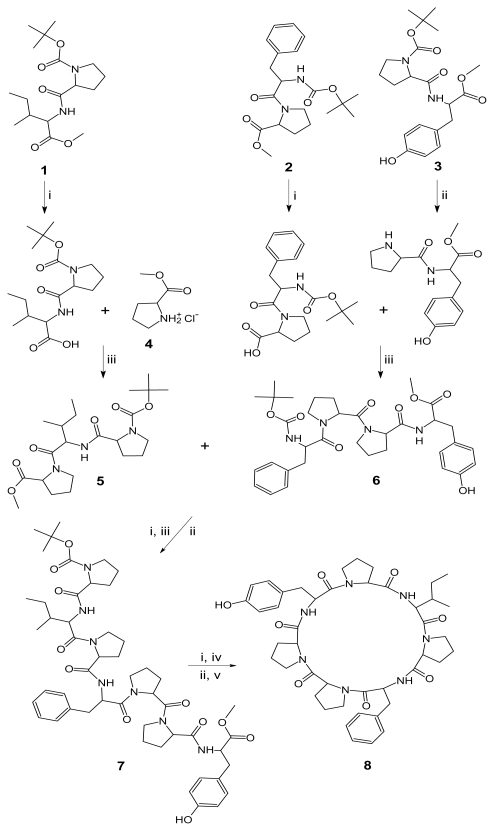
Synthetic route for natural cycloheptapeptide stylisin 2 (**8**).

In present study, a disconnection strategy was employed to carry out the first total synthesis of stylisin 2. The cyclic heptapeptide molecule was split into three dipeptide units Boc-l-Pro-l-Ile-OMe (**1**), Boc-l-Phe-l-Pro-OMe (**2**), Boc-l-Pro-l-Tyr-OMe (**3**) and single amino acid unit l-Pro-OMe·HCl (**4**). The required dipeptides **1**–**3** were prepared by coupling of Boc-amino acids, *viz*. Boc-l-Pro and Boc-l-Phe with the corresponding amino acid methyl ester hydrochlorides *viz.* l-Ile-OMe·HCl, l-Pro-OMe·HCl and l-Tyr-OMe·HCl employing dicyclohexylcarbodiimide (DCC) as coupling agent [28]. Racemization was suitably controlled during normal peptide couplings by the use of less strenuous conditions, *i.e.*, low temperature, controlled quantity of the base such as triethylamine (TEA) and a suitable solvent like dimethylformamide (DMF) or tetrahydrofuran (THF). The ester group of dipeptide **1** was removed by alkaline hydrolysis with LiOH and the deprotected peptide was coupled with amino acid methyl ester hydrochloride **4** using diisopropylcarbodiimide (DIPC)/DCC and triethylamine (TEA), to get the tripeptide unit Boc-l-Pro-l-Ile-l-Pro-OMe (**5**). Similarly, dipeptide **2** after deprotection at the carboxyl end, was coupled with dipeptide **3** deprotected at amino terminal, to get the tetrapeptide unit Boc-l-Phe-l-Pro-l-Pro-l-Tyr-OMe (**6**). After removal of the ester group of tripeptide **5** and the Boc group of tetrapeptide **6**, these deprotected units were coupled to get the linear heptapeptide unit Boc-l-Pro-l-Ile-l-Pro-l-Phe-l-Pro-l-Pro-l-Tyr-OMe (**7**). The methyl ester group of the linear peptide fragment was replaced by a pentafluorophenyl (pfp)/*p*-nitrophenyl (pnp) ester group. The Boc-group of the resulting compound was removed using trifluoroacetic acid (TFA) and the deprotected linear fragment was now cyclized by keeping the whole contents at 0 °C for 7 days in the presence of catalytic amounts of TEA/NMM/pyridine to get cyclooligopeptide **8** (Scheme 1). The cyclization of the linear peptide was indicated by the disappearance of absorption bands at 1,750, 1,271 cm^−1^ and 1,389, 1,373 cm^−1^ (CO stretching of ester and CH deformation of *tert*-butyl group) in the IR spectrum of compound **8**. Formation of cyclooligopeptide was further confirmed by the disappearance of the singlet at δ 1.49 corresponding to the nine protons of the Boc *tert*-butyl group and the singlet at d 3.54 corresponding to the three protons of the methyl ester in the ^1^H-NMR spectrum of compound **8**. Seven signals between δ 4.39–3.85 in the proton spectrum of compound **8** agree with the expected peptidic structure for the compound, with these signals being attributable to the alpha protons of seven amino acids. The NMR data of the synthetic stylisin 2 are identical with those of the natural product within the error range of d 0.07 (^1^H) and 1 (^13^C), respectively. Furthermore, the ^1^H-NMR and ^13^C-NMR spectra of the synthesized cycloheptapeptide showed characteristic peaks confirming the presence of all the 57 protons and 44 carbon atoms. The presence of a [M + H]^+^ ion peak at *m/z* 812.9 corresponding to the molecular formula C_44_H_57_N_7_O_8_ in the ESI-MS/MS spectrum of compound **8**, along with other fragment ion peaks resulting from cleavage at the ‘Ile-Pro’ and ‘Phe-Pro’ amide bond levels, showed the exact sequence of attachment of all the seven amino acid moieties in a chain. In addition, elemental analysis of compound **8** afforded values (±0.03) in accordance with the molecular composition. Synthesis of stylisin 2 (**8**) was thus carried out successfully with good yield and pyridine was proven to be an effective base for cyclization of the linear heptapeptide fragment.

### Reagents and conditions

(i) LiOH, THF-H_2_O (1:1), RT, 1 h; (ii) TFA, CHCl_3_, RT, 1 h; (iii) DIPC/DCC, TEA, DMF/THF, RT, 24–36 h; (iv) DCC, pfp/pnp, RT, 12 h; (v) TEA/NMM/pyridine, CHCl_3_, 7 days, 0 °C.

Comparison of antimicrobial screening data (Table 1) suggested that cyclooligopeptide **8** exhibited higher antibacterial activity against the Gram negative bacteria *Klebsiella pneumoniae* and *Pseudomonas aeruginosa*, and potent antifungal activity against pathogenic *Candida albicans* with MIC values of 12.5–6 μg/mL, in comparison to a standard drug—gatifloxacin. Moreover, compound **8** displayed a moderate level of biological activity against the dermatophytes *Microsporum audouinii* and *Trichophyton mentagrophytes* with MIC values of 6 μg/mL. However, compound **8** displayed no significant activity neither against Gram positive bacteria nor against the fungus *A. niger.*

Analysis of the antimicrobial activity data revealed that the linear heptapeptide **7** displayed less bioactivity against pathogenic bacteria and fungi compared to its cyclic form **8**. This is because cyclization of peptides reduces the degree of freedom for each constituent within the ring and thus leads to substantially reduced flexibility, increased potency and selectivity for cyclic peptides. Further, the inherent flexibility of linear peptides leads to different conformations which can bind to more than one receptor molecules, resulting in undesirable adverse effects.

## 3. Experimental

Melting points were determined by open capillary method and are uncorrected. l-Amino acids, di-*tert*-butylpyrocarbonate (Boc_2_O), DCC, DIPC, TFA, TEA, pyridine and NMM were procured from SpectroChem Limited (Mumbai, India). IR spectra were recorded on a Shimadzu 8700 FTIR spectrophotometer using a thin film supported on KBr pellets or utilizing chloroform solutions. ^1^H-NMR and ^13^C-NMR spectra were recorded on a Bruker AC NMR spectrometer (300 MHz) using CDCl_3_ as solvent and TMS as internal standard. The mass spectra were recorded on a JMS-DX 303 Mass spectrometer (Jeol, Tokyo, Japan) operating at 70 eV in ESI-MS/MS mode. Optical rotation of the synthesized peptide derivatives was measured in methanol on an automatic polarimeter in a 2 dm tube at 25 °C using a sodium lamp. Elemental analyses of all compounds were performed on a Vario EL III elemental analyzer. Purity of all synthesized compounds was checked by TLC on precoated silica gel G plates utilizing chloroform/methanol in different ratios (8:2/7:3, *v/v*) as developing solvent system and spots were detected by exposure to iodine vapours in a tightly closed chamber.

### 3.1. General procedure for the preparation of linear tri/tetrapeptide segments

To a solution of amino acid methyl ester hydrochloride or dipeptide methyl ester (0.01 mol) in DMF (25 mL), TEA (0.021 mol) was added in portions at 0 °C and the reaction mixture was stirred for 15 min, maintaining the temperature between 0–5 °C. Boc-dipeptide (0.01 mol) was dissolved in DMF (25 mL) and DIPC or DCC (0.01 mol) was added in portions with stirring. Stirring was first done for 1 h at 0–5 °C and then further for 24 h at room temperature (RT). Precaution was taken not to allow the reaction temperature to exceed RT to avoid the risk of racemization. After the completion of the reaction, the reaction mixture was diluted with an equal amount of water. The precipitated solid was filtered, washed with water and recrystallized from a mixture of chloroform and petroleum ether (bp 40–60 °C) followed by cooling at 0 °C to get the title compounds.

#### tert-Butyloxycarbonyl-l-prolyl-l-isoleucyl-l-proline methyl ester (**5**)

Semisolid mass; Yield 87%; [α]_D_ –41.4°; R_f_ 0.78; IR (CHCl_3_): *v* 3,122 (N–H str, amide), 2,997–2,989 (C–H str, CH_2_, Pro), 2,965, 2,927 (C–H str, asym, CH_3_ and CH_2_), 2,869 (C–H str, sym, CH_3_), 1,752 (C=O str, ester), 1,678, 1,673, 1,644 (C=O str, 3° and 2° amide), 1,536 (N–H bend, 2° amide), 1,388, 1,375 (C–H bend, *tert*-butyl), 1,269 (C–O str, ester) cm^−1^; ^1^H-NMR (CDCl_3_): δ 6.25 (1H, br. s, NH), 4.52 (1H, t, *J* = 8.6 Hz, H-α, Ile), 4.11 (1H, t, *J* = 6.9 Hz, H-α, Pro-1), 3.92 (1H, t, *J* = 6.9 Hz, H-α, Pro-2), 3.62 (3H, s, OCH_3_), 3.41 (2H, t, *J* = 7.2 Hz, H-δ, Pro-2), 3.21 (2H, t, *J* = 7.1 Hz, H-δ, Pro-1), 2.55 (2H, q, H-β, Pro-1), 2.04 (3H, m, H-β, Pro-2 and H-β, Ile), 1.97 (2H, m, H-γ, Pro-2), 1.90 (2H, m, H-γ, Pro-1), 1.64 (2H, q, H-γ, Ile), 1.48 (9H, s, *tert*-butyl), 1.03 (3H, d, *J* = 5.9 Hz, H-γ′, Ile), 0.97 (3H, t, *J* = 7.8 Hz, H-δ, Ile); Anal. Calcd. for C_32_H_37_N_3_O_6_: C, 60.12; H, 8.48; N, 9.56. Found: C, 60.15; H, 8.49; N, 9.54%.

#### tert-Butyloxycarbonyl-l-phenylalanyl-l-prolyl-l-prolyl-l-tyrosine methyl ester (**6**)

Semisolid mass; Yield 79%; [α]_D_ –69.2°; R_f_ 0.59; IR (CHCl_3_): *v* 3,372 (O–H str, Tyr), 3,123, 3,119 (N–H str, amide), 3,069, 3,055 (C–H str, rings), 2,999–2,987 (C–H str, CH_2_, Pro), 2,929, 2,925 (C–H str, asym, CH_2_), 2,875, 2,852 (C–H str, sym, CH_3_ and CH_2_), 1,748 (C=O str, ester), 1,682–1,678, 1,645, 1,639 (C=O str, 3° and 2° amide), 1,588, 1,483 (skeletal bands), 1,539, 1,532 (N–H bend, 2° amide), 1,389, 1,374 (C–H bend, *tert*-butyl), 1,266 (C–O str, ester), 1,230 (C–O str, phenolic), 828, 725, 689 (C–H bend, out-of-plane (oop), rings) cm^−1^; ^1^H-NMR (CDCl_3_): δ 8.65 (1H, br. s, NH), 7.50 (2H, t, *J* = 7.2 Hz, 4.4 Hz, H at C-3 and C-5, Phe), 6.94 (1H, t, *J* = 6.2 Hz, H at C-4, Phe), 6.90 (2H, d, *J* = 8.5 Hz, H at C-2 and C-6, Tyr), 6.85 (2H, d, *J* = 8.8 Hz, 5.4 Hz, H at C-2 and C-6, Phe), 6.79 (2H, d, *J* = 8.6 Hz, H at C-3 and C-5, Tyr), 6.44 (1H, br. s, NH), 5.95 (1H, br. s, OH), 5.06 (1H, q, *J* = 7.9 Hz, H-α, Tyr), 4.60 (1H, q, *J* = 5.5 Hz, H-α, Phe), 4.55 (1H, t, *J* = 6.9 Hz, H-α, Pro-1), 4.39 (1H, t, *J* = 6.9 Hz, H-α, Pro-2), 3.68 (4H, m, H-δ, Pro-1 and Pro-2), 3.53 (3H, s, OCH_3_), 2.99 (4H, m, H-β, Phe and Tyr), 2.67 (4H, m, H-β, Pro-1 and Pro-2), 1.92 (4H, m, H-γ, Pro-1 and Pro-2), 1.55 (9H, s, *tert*-butyl); Anal. Calcd. for C_34_H_44_N_4_O_8_: C, 64.14; H, 6.96; N, 8.80. Found: C, 64.15; H, 6.99; N, 8.79%.

### 3.2. Deprotection of tripeptide unit at the carboxyl terminal

To a solution of the tripeptide **5** (4.4 g, 0.01 mol) in THF-H_2_O (1:1, 36 mL), 0.36 g (0.015 mol) of LiOH was added at 0 °C. The mixture was stirred at room temperature for 1 h and then acidified to pH 3.5 with 1 N H_2_SO_4_. The aqueous layer was extracted with Et_2_O (3 × 25 mL). The combined organic extracts were dried over anhydrous Na_2_SO_4_ and concentrated under reduced pressure. The crude product was finally crystallized from methanol and ether to afford pure deprotected compound.

### 3.3. Deprotection of tetrapeptide unit at amino terminal

Tetrapeptide **6** (6.36 g, 0.01 mol) was dissolved in CHCl_3_ (15 mL) and treated with CF_3_COOH (2.28 g, 0.02 mol). The resulting solution was stirred at room temperature for 1 h, washed with saturated NaHCO_3_ solution (25 mL). The organic layer was dried over anhydrous Na_2_SO_4_ and concentrated under reduced pressure. The crude product was purified by crystallization from CHCl_3_ and petroleum ether (bp 40–60 °C) to give the pure deprotected tetrapeptide unit.

### 3.4. Procedure for the synthesis of the linear heptapeptide unit

Deprotected tetrapeptide (5.36 g, 0.01 mol) was dissolved in tetrahydrofuran (THF, 35 mL). To this solution, TEA (2.8 mL, 0.021 mol) was added in portions at 0 °C and the resulting mixture was stirred for 15 min. Deprotected tripeptide (4.25 g, 0.01 mol) was dissolved in THF (35 mL) and DIPC (1.26 g, 0.01 mol) was added in portions to the above mixture with stirring, maintaining the temperature between 0–10 °C. Stirring was continued at room temperature for 36 h, after which the reaction mixture was filtered and the filtrate was washed with 5% NaHCO_3_ and saturated NaCl solutions (30 mL each). The organic layer was dried over anhydrous Na_2_SO_4_, filtered and evaporated in vacuum. The crude product was recrystallized from a mixture of chloroform and petroleum ether (bp 40–60 °C) followed by cooling at 0 °C.

#### tert-Butyloxycarbonyl-l-prolyl-l-isoleucyl-l-prolyl-l-phenylalanyl-l-prolyl-l-prolyl-l-tyrosine methyl ester (**7**)

Semisolid mass; Yield 83%; [α]_D_ –52.7°; R_f_ 0.85; IR (CHCl_3_): *v* 3,375 (O–H str, Tyr), 3,128–3,123 (N–H str, amide), 3,065 (C–H str, ring), 2,999–2,986 (C–H str, CH_2_, Pro), 2,968–2,963, 2,927–2,922 (C–H str, asym, CH_3_ and CH_2_), 2,876–2,872, 2,858–2,853 (C–H str, sym, CH_3_ and CH_2_), 1,750 (C=O str, ester), 1,679–1,673, 1,645 (C=O str, 3° and 2° amide), 1,589, 1,482 (skeletal bands), 1539, 1,534 (N–H bend, 2° amide), 1,389, 1,373 (C–H bend, *tert*-butyl), 1,271 (C–O str, ester), 1,233 (C–O str, phenolic), 829, 722, 687 (C–H bend, out-of-plane (oop), rings) cm^−1^; ^1^H-NMR (CDCl_3_): δ 9.85 (1H, br. s, NH), 8.62 (1H, br. s, NH), 7.17 (2H, dd, *J* = 7.2 Hz, 4.4 Hz, H at C-3 and C-5, Phe), 7.01 (1H, t, *J* = 6.2 Hz, H at C-4, Phe), 6.89, 6.81 (2H each, A2B2, *J* = 8.5 Hz, H at C-2, 6, 3 and 5, Tyr), 6.84 (2H, dd, *J* = 8.7 Hz, 5.3 Hz, H at C-2 and C-6, Phe), 6.25 (1H, br. s, NH), 5.97 (1H, br. s, OH), 5.11 (1H, m, H-α, Phe), 5.04 (1H, q, H-α, Tyr), 4.38 (1H, t, *J* = 6.9 Hz, H-α, Pro-2), 4.33 (1H, t, *J* = 8.6 Hz, H-α, Ile), 4.16 (1H, t, *J* = 6.9 Hz, H-α, Pro-1), 4.09 (2H, m, H-α, Pro-1 and Pro-2), 3.66 (2H, t, *J* = 7.1 Hz, H-δ, Pro-4), 3.54 (3H, s, OCH_3_), 3.33 (4H, m, H-δ, Pro-2 and Pro-3), 3.21 (2H, t, *J* = 7.2 Hz, H-δ, Pro-1), 3.01 (4H, m, H-β, Phe and Tyr), 2.68 (6H, m, H-β, Pro-2, Pro-3 and Pro-4), 2.54 (2H, q, H-β, Pro-1), 2.04 (1H, m, H-β, Ile), 1.92 (8H, m, H-δ, Pro-1-4), 1.64 (2H, m, H-γ, Ile), 1.49 (9H, s, *tert*-butyl), 1.00 (3H, d, *J* = 5.9 Hz, H-γ′, Ile), 0.96 (3H, t, *J* = 7.8 Hz, H-δ, Ile); Anal. Calcd. for C_50_H_69_N_7_O_11_: C, 63.61; H, 7.37; N, 10.38. Found: C, 63.60; H, 7.39; N, 10.40%.

### 3.5. Synthesis of the cyclic heptapeptide **8**

To synthesize stylisin 2 **(8**), linear heptapeptide unit **7** (4.72 g, 0.005 mol) was deprotected at carboxyl end using LiOH (0.18 g, 0.0075 mol) to get Boc-l-prolyl-l-isoleucyl-l-prolyl-l-phenylalanyl-l-prolyl-l-prolyl-l-tyrosine-OH. The deprotected heptapeptide unit (4.65 g, 0.005 mol) was now dissolved in CHCl_3_ (50 mL) at 0 °C. To this solution, *p*-nitrophenol or pentafluorophenol (0.94 g or 1.23 g, 0.0067 mol) and DIPC (0.63 g, 0.005 mol) were added and the mixture was stirred at room temperature for 12 h. The reaction mixture was filtered and the filtrate was washed with 10% NaHCO_3_ solution (3 × 25 mL) and finally washed with 5% HCl (2 × 30 mL) to get the corresponding *p*-nitro-phenyl or pentafluorophenyl ester Boc-l-prolyl-l-isoleucyl-l-prolyl-l-phenylalanyl-l-prolyl-l-prolyl-l-tyrosine-Opnp or Boc-l-prolyl-l-isoleucyl-l-prolyl-l-phenylalanyl-l-prolyl-l-prolyl-l-tyrosine-Opfp. To this compound (4.2 g or 4.38 g, 0.004 mol) dissolved in CHCl_3_ (35 mL), CF_3_COOH (0.91 g, 0.008 mol) was added, stirred at room temperature for 1 h and washed with 10% NaHCO_3_ solution (2 × 25 mL). The organic layer was dried over anhydrous Na_2_SO_4_ to get l-prolyl-l-isoleucyl-l-prolyl-l-phenylalanyl-l-prolyl-l-prolyl-l-tyrosine-Opnp or l-prolyl-l-isoleucyl-l-prolyl-l-phenylalanyl-l-prolyl-l-prolyl-l-tyrosine-Opfp, which was dissolved in CHCl_3_ (25 mL) and TEA/NMM/pyridine (2.8 mL or 2.21 mL or 1.61 mL, 0.021 mol) was added. Then, above alkaline solution was kept at 0 °C for 7 days. The reaction mixture was washed with 10% NaHCO_3_ (3 × 25 mL) and 5% HCl (2 × 30 mL) solutions. The organic layer was dried over anhydrous Na_2_SO_4_ and crude cyclized product was crystallized from CHCl_3_/*n*-hexane to get pure cyclic product **8**.

#### Cyclo(l-prolyl-l-isoleucyl-l-prolyl-l-phenylalanyl-l-prolyl-l-prolyl-l-tyrosinyl) (**8**)

White solid; Yield 89% (3.61 g, C_5_H_5_N), 82% (3.33 g, NMM), 79% (3.21 g, TEA); mp 216 °C (dec); [α]_D_ –39.8° (*c* 0.2, MeOH) (−39.9 for natural stylisin 2); R_f_ 0.68 (CHCl_3_:AcOH:H_2_O, 3:2:5); IR (KBr): *v* 3,377 (O–H str, Tyr), 3,129, 3,125-3,121 (N–H str, amide), 3,069–3,066 (C–H str, ring), 2,998–2,985 (C–H str, CH_2_, Pro), 2,969–2,965, 2,929, 2,925 (C–H str, asym, CH_3_ and CH_2_), 2,875–2,872, 2,859–2,855 (C–H str, sym, CH_3_ and CH_2_), 1,676–1,672, 1,645–1,640 (C=O str, 3° and 2° amide), 1,587, 1,480 (skeletal bands), 1,537, 1,533–1,530 (N–H bend, 2° amide), 1,236 (C–O str, phenolic), 828, 720, 689 (C–H bend, out-of-plane (oop), rings) cm^−1^; ^1^H-NMR (CDCl_3_): δ 9.75 (1H, br. s, NH), 9.72 (1H, br. s, NH), 8.52 (1H, br. s, NH), 7.18 (2H, dd, *J* = 7.2 Hz, 4.4 Hz, H at C-3 and C-5, Phe), 7.03 (1H, t, *J* = 6.2 Hz, H at C-4, Phe), 6.97, 6.90 (2H each, A2B2, *J* = 8.5 Hz, H at C-2, 6, 3 and 5, Tyr), 6.85 (2H, d, *J* = 8.6 Hz, 5.4 Hz, H at C-2 and C-6, Phe), 5.95 (1H, br. s, OH), 4.39 (2H, m, H-α, Phe and Tyr), 4.25 (1H, t, *J* = 7.0 Hz, H-α, Pro-4), 4.21 (1H, t, *J* = 6.9 Hz, H-α, Pro-3), 3.91 (2H, m, H-α, Pro-1 and Pro-2), 3.85 (1H, t, *J* = 8.6 Hz, H-α, Ile), 3.60 (2H, t, *J* = 7.2 Hz, H-δ, Pro-4), 3.26 (6H, m, H-δ, Pro-1–3), 2.67 (8H, m, H-β, Pro-1–4), 2.61 (4H, m, H-β, Phe and Tyr), 1.83 (8H, m, H-δ, Pro-1–4), 1.61 (2H, m, H-γ, Ile), 1.55 (1H, m, H-β, Ile), 1.00 (3H, d, *J* = 5.9 Hz, H-γ′, Ile), 0.95 (3H, t, *J* = 7.8 Hz, H-δ, Ile); ^13^C-NMR (CDCl_3_): δ 173.5 (C=O, Ile), 172.8, 171.6 (2C, C=O, Pro-2 and Pro-4), 170.9 (C=O, Pro-1), 170.3, 169.9 (2C, C=O, Phe and Tyr), 169.3 (C=O, Pro-3), 153.9 (C-*p*, Tyr), 138.5, 133.4 (2C, C-γ, Phe and Tyr), 132.1 (2C, C-*o*, Phe), 129.5 (2C, C-*o*, Tyr), 128.9 (2C, C-*m*, Phe), 128.3 (2C, C-*m*, Tyr), 128.0 (C-*p*, Phe), 58.7 (2C, C-α, Pro-1 and Pro-2), 57.3, 55.7 (2C, C-α, Pro-4 and Pro-3), 54.3 (C-α, Ile), 53.6, 52.8 (2C, C-α, Phe and Tyr), 48.9, 48.2 (2C, C-δ, Pro-1 and Pro-3), 46.6, 45.8 (2C, C-δ, Pro-2 and Pro-4), 43.2 (C-β, Tyr), 37.8 (C-β, Phe), 35.6 (C-β, Ile), 34.4, 33.9 (2C, C-β, Pro-1 and Pro-4), 32.2, 30.5 (2C, C-β, Pro-2 and Pro-3), 24.8, 23.6 (2C, C-γ, Ile and Pro-3), 23.2, 22.6, 21.7 (3C, C-γ, Pro-1, Pro-2 and Pro-4), 16.2 (C-γ′, Ile), 10.3 (C-δ, Ile); ESI-MS/MS *m/z* [RI]: 812.9 [(M + 1)^+^, 100], 784.9 [(812.9 – CO)^+^, 14], 699.8 [(H-Pro-Phe-Pro-Pro-Tyr-Pro)^+^, 37], 671.8 [(699.8 – CO)^+^, 11], 665.8 [(H-Pro-Pro-Tyr-Pro-Ile-Pro)^+^, 29], 602.7 [(H-Pro-Phe-Pro-Pro-Tyr)^+^, 64], 574.7 [(602.7 – CO)^+^, 19], 455.5 [(H-Pro-Pro-Tyr-Pro)^+^, 38], 439.5 [(H-Pro-Phe-Pro-Pro)^+^, 49], 411.5 [(439.5 – CO)^+^, 21], 358.4 [(H-Pro-Pro-Tyr)^+^, 66], 342.4 [(H-Pro-Phe-Pro)^+^, 32], 330.4 [(358.4 – CO)^+^, 11], 245.3 [(H-Pro-Phe)^+^, 25], 217.2 [(245.3 – CO)^+^, 11], 195.2 [(H-Pro-Pro)^+^, 17], 136.1 [Tyr (C_8_H_10_NO)^+^, 14], 120.1 [Phe (C_8_H_10_N)^+^, 10], 107.1 [(C_7_H_7_O)^+^, 12], 98.1 [(Pro)^+^, 15], 93.1 [(C_6_H_5_O)^+^, 9], 91.1 [(C_7_H_7_)^+^, 11], 86.1 [Ile (C_5_H_12_N)^+^, 12], 77.1 [(C_6_H_5_)^+^, 7], 70.1 [H-Pro (C_4_H_8_N)^+^, 23], Ile: 57.1 [(C_4_H_9_)^+^, 6], 29.1 [(C_2_H_5_)^+^, 9], 15.0 [(CH_3_)^+^, 13]; Anal. Calcd. for C_44_H_57_N_7_O_8_: C, 65.09; H, 7.08; N, 12.08. Found: C, 65.11; H, 7.05; N, 12.10%.

### 3.6. Biological activity studies

#### 3.6.1. Antibacterial screening

Newly synthesized linear and cyclic heptapeptides **7** and **8** were evaluated for their antibacterial potential against two Gram-positive bacteria—*Bacillus subtilis* and *Staphylococcus aureus*—and two Gram-negative bacteria—*Pseudomonas aeruginosa* and *Klebsiella pneumonia*—at concentrations of 50–6.25 μg/mL by using a modified Kirby-Bauer disc diffusion method [29]. MIC values of the test compounds were determined by the Tube Dilution Technique. Both linear and cyclic heptapeptides were dissolved separately using DMF to prepare a stock solution of 1 mg/mL. Stock solution was aseptically transferred and suitably diluted with sterile broth medium to contain seven different concentrations of each test compound ranging from 200–3.1 μg/mL in different test tubes. All the tubes were inoculated with one loopful of one of the test bacteria. The process was repeated with different test bacteria and different samples. Tubes inoculated with bacterial cultures were incubated at 37 °C for 18 h and the presence/absence of growth of the bacteria was observed. From these results, MIC of each test compound was determined against each test bacterium. A possible spore suspension was prepared in sterile distilled water from 5 days old culture of the test bacteria growing on nutrient broth media. About 20 mL of the growth medium was transferred into sterilized Petri plates and inoculated with 1.5 mL of spore suspension (spore concentration ~6 × 10^4^ spores/mL). Filter paper disks of 6 mm diameter and 1 mm thickness were sterilized by autoclaving at 121 °C (15 psi) for 15 min. Each Petri plate was divided into five equal portions along the diameter to place one disc. Three discs of test sample were placed on three portions together with one disc with reference drug—gatifloxacin and a disk impregnated with the solvent (DMF) as negative control. The Petri plates inoculated with bacterial cultures were incubated at 37 °C for 18 h. Diameters of the inhibition zones (in mm) were measured and the average diameters for test sample were calculated in triplicate. The diameters obtained for the test sample were compared with that produced by the standard drug. The results of antibacterial studies are presented in Table 1.

#### 3.6.2. Antifungal screening

Serial plate dilution method was used for the evaluation of antifungal activity against the diamorphic fungal strain *C. albicans* and three other fungal strains, including *A. niger* and two cutaneous fungal strains, *M. audouinii* and *T. mentagrophytes*, at concentrations of 50–6.25 μg/mL [30]. MIC values of the test compounds were determined by employing the same technique as used for antibacterial studies using DMSO instead of DMF and tubes inoculated with fungal cultures were incubated at 37 °C for 48 h. After incubation, the presence/absence of fungal growth was observed and MIC of test compounds was determined against each test fungus. A spore suspension in normal saline (0.91% *w/v* of NaCl) was prepared from culture of the test fungi on Sabouraud’s broth media. After transferring growth medium, Petri plates were inoculated with spore suspension. After drying, wells were made using an agar punch and test samples, reference drug—griseofulvin and negative control (DMSO) were placed in labeled wells in each Petri plate. The Petri plates inoculated with fungal cultures were incubated at 37 °C for 48 h. Antifungal activity was determined by measuring the diameter of the inhibition zone for triplicate sets. Activity of each compound was compared with reference standard. The results of antifungal studies are given in the Table 1.

## 4. Conclusions

The first total synthesis of the natural peptide stylisin 2 (**8**) was accomplished in good yield via coupling reactions utilizing different carbodiimides. Diisopropylcarbodiimide (DIPC)/TEA coupling method proved to be yield-effective, in comparison to methods utilizing DCC/TEA or NMM, providing 10–12% better yield. A pentafluorophenyl ester proved to be better for the activation of the acid functionality of the linear heptapeptide unit when compared to *p*-nitrophenol. Pyridine was found to be a good base for intramolecular cyclization of the linear peptide fragment in comparison to TEA and NMM. The synthesized cycloheptapeptide displayed high antibacterial activity against *K. pneumoniae* and *P. aeruginosa*, along with potent antifungal activity against *C. albicans*. In comparison, Gram negative bacteria were found to be more sensitive than Gram positive bacteria towards the newly synthesized peptide. On passing toxicity tests, synthesized cyclooligopeptide **8** may prove good candidate for clinical studies and can be new antimicrobial drug of future.

## Figures and Tables

**Table 1:** Antimicrobial activity data for compound **7** and **8**.

Compound	Diameter of zone of inhibition (mm)							
	*Bacterial strains*				*Fungal strains*			

	*B. sub.*	*S. aur.*	*P. aeru.*	*K. pneu.*	*C. alb.*	*M. audo.*	*A. niger*	*T. menta.*
**7**	10(25)†	14(12.5)	22(6)	23(6)	21(6)	11(6)	–	14(6)
**8**	13(25)	16(12.5)	26(6)	28(6)	27(6)	15(6)	–	18(6)
Control *	–	–	–	–	–	–	–	–
Gatifloxacin	18(12.5)	28(6)	22(6)	25(6)	–	–	–	–
Griseofulvin	–	–	–	–	20(6)	17(6)	18(12.5)	20(6)

B. sub.: Bacillus subtilis; S. aur.: Staphylococcus aureus; P. aeru.: Pseudomonas aeruginosa; K. pneu.: Klebsiella pneumoniae; C. alb.: Candida albicans; M. audo.:Microsporum audouinii; A. niger: Aspergillus niger; T. menta.: Trichophyton mentagrophytes.

† Values in bracket are MIC values (μg/mL);

* DMF/DMSO.
